# Antiangiogenic Therapy for Diabetic Nephropathy

**DOI:** 10.1155/2017/5724069

**Published:** 2017-08-01

**Authors:** Katsuyuki Tanabe, Yohei Maeshima, Yasufumi Sato, Jun Wada

**Affiliations:** ^1^Department of Nephrology, Rheumatology, Endocrinology and Metabolism, Okayama University Graduate School of Medicine, Dentistry and Pharmaceutical Sciences, Okayama 700-8558, Japan; ^2^Department of Vascular Biology, Institute of Development, Aging and Cancer, Tohoku University, Sendai 980-8575, Japan

## Abstract

Angiogenesis has been shown to be a potential therapeutic target for early stages of diabetic nephropathy in a number of animal experiments. Vascular endothelial growth factor (VEGF) is the main mediator for abnormal angiogenesis in diabetic glomeruli. Although beneficial effects of anti-VEGF antibodies have previously been demonstrated in diabetic animal experiments, recent basic and clinical evidence has revealed that the blockade of VEGF signaling resulted in proteinuria and renal thrombotic microangiopathy, suggesting the importance of maintaining normal levels of VEGF in the kidneys. Therefore, antiangiogenic therapy for diabetic nephropathy should eliminate excessive glomerular angiogenic response without accelerating endothelial injury. Some endogenous antiangiogenic factors such as endostatin and tumstatin inhibit overactivation of endothelial cells but do not specifically block VEGF signaling. In addition, the novel endothelium-derived antiangiogenic factor vasohibin-1 enhances stress tolerance and survival of the endothelial cells, while inhibiting excess angiogenesis. These factors have been demonstrated to suppress albuminuria and glomerular alterations in a diabetic mouse model. Thus, antiangiogenic therapy with promising candidates will possibly improve renal prognosis in patients with early stages of diabetic nephropathy.

## 1. Introduction

Diabetic nephropathy has become a leading cause of end-stage kidney disease (ESKD) in developed countries. The global pandemic of obesity will further result in the increased prevalence of diabetic nephropathy. The current mainstay of the treatment of diabetic nephropathy is glycemic control, as well as lowering blood pressure with specific classes of antihypertensive agents that block renin-angiotensin-aldosterone system (RAAS). RAAS inhibitors have been demonstrated to have renoprotective effects in patients with diabetic nephropathy, but their efficacies have not always been determined to be sufficient in clinical practice. In the same way, intensive glycemic control resulted in inconsistent benefits in patient with nephropathy among large clinical trials. Thus, once overt diabetic nephropathy develops, specific therapies targeting the underlying mechanisms are required in order to prevent the progression to ESKD, in addition to blood pressure control with RAAS inhibitors and appropriate glycemic control.

Angiogenesis is one of the potential targets for the treatment of diabetic nephropathy. Vascular endothelial growth factor (VEGF) is a critical regulator of angiogenesis, and its glomerular expression is involved in the pathogenesis of diabetic nephropathy. Antiangiogenic (in particular, anti-VEGF) therapy for diabetic nephropathy has been shown to be a promising strategy in many animal experiments, but some recent evidence raises concerns about its use in clinical practice. In this review, we will outline abnormal angiogenesis and VEGF in the pathogenesis of diabetic nephropathy, explain the benefits and limitations of antiangiogenic therapy, and then finally propose alternative antiangiogenic strategies to address such concerns.

## 2. Roles of VEGF in Angiogenesis

Angiogenesis is the physiological and pathological process through which new blood vessels develop from preexisting vessels. It is involved in embryogenesis, in wound healing, in tumor growth and metastasis, in atherosclerosis, and in the onset of inflammatory diseases in humans [1]. A number of proangiogenic and antiangiogenic factors are responsible for regulating angiogenesis, including VEGF, basic fibroblast growth factor (bFGF), angiopoietins, and ephrin.

VEGF is one of the most potent proangiogenic factors. The VEGF family consists of VEGF-A, VEGF-B, VEGF-C, VEGF-D, and placental growth factor (PlGF) in mammals [2]. VEGF-A is a prototype member of the family and is crucially involved in physiological and pathological angiogenesis. VEGF-A shows haploid insufficiency, as inactivation of a single copy of the gene resulted in embryonic lethality in mice due to immature organ development, including impaired blood vessel formation [3, 4], suggesting an essential role of VEGF-A in vasculogenesis and angiogenesis. VEGF-A has a variety of functions: though perhaps most importantly, it promotes angiogenesis through stimulation of the proliferation and migration of endothelial cells [5]. VEGF-A also has vascular permeability activity and monocyte chemotactic activity [6, 7], which are involved in inflammation in some pathological processes. There are several isoforms of VEGF-A through alternative splicing, such as VEGF-A_121_, VEGF-A_165_, VEGF-A_165_b, VEGF-A_189_, and VEGF-A_206_ in humans [8–10]. Among isoforms of VEGF-A, VEGF-A_165_ is quantitatively and qualitatively predominant.

VEGF-A binds to and activates the tyrosine kinase receptors, VEGFR-1 (Flt-1) and VEGFR-2 (KDR/Flk-1) [2]. VEGFR-1 has a much higher affinity for VEGF-A, whereas VEGFR-2 has approximately 10-fold higher tyrosine kinase activity [11]. Therefore, angiogenic signals are mainly generated from VEGF-A bound to VEGFR-2, whereas VEGFR-1 could play as negative regulator of VEGF-A at least in some conditions, such as embryogenesis. VEGF-A-bound VEGFR-2 undergoes dimerization and tyrosine phosphorylation, and this reaction promotes the phosphorylation of several targets, including phosphoinositide 3-kinase (PI3K) and Ras GTPase-activating proteins [12]. Phospholipase C*γ* is also activated in VEGF-A-bound VEGFR-2, followed by the activation of protein kinase C (PKC), especially PKC*β* [13, 14]. PKC then activates Raf-MEK-extracellular signal-regulated (ERK) pathways for endothelial cell proliferation [14]. In addition, the activation of VEGFR-2 inhibits apoptosis of endothelial cells via the PI3K-Akt pathway [15]. VEGF-A also binds to neuropilin 1 (NRP1), which presents VEGF-A to VEGFR-2 and enhances VEGF-A-induced VEGFR-2 signaling [16]. Compared with VEGF-A_165_, VEGF-A_165_b does not fully activate VEGFR-2 and thus could act as a much weaker agonist for VEGFR-2 than VEGF-A_165_ [17]. This is potentially explained by the fact that VEGF-A_165_b has distinct C-terminal amino acid sequence, which leads to insufficient interaction with NRP1 [18]. VEGF-A_121_ could bind to NRP1, with lower affinity than VEGF-A_165_ [19]. On the other hand, VEGF-A_189_ has higher affinity for NRP1 than VEGF-A_165_ [20]. However, secreted VEGF-A_189_ could be trapped by extracellular matrix through its highly basic sequences [21]. Therefore, VEGF-A_165_ has the most potent agonist activity for VEGFR-2 in vivo.

## 3. Angiogenesis in Diabetic Nephropathy

Diabetic nephropathy is clinically defined by the presence of microalbuminuria followed by a progressive increase in proteinuria and a decrease in glomerular filtration rate (GFR) in the setting of long-standing diabetes with or without other microangiopathies, such as retinopathy. An earlier finding of (preclinical) diabetic nephropathy is glomerular hyperfiltration seen as increased GFR, with no morphological changes or only glomerular hypertrophy. Histologically, glomerular alterations in diabetic nephropathy include glomerular basement membrane thickening and/or mesangial matrix accumulation in the early stages and Kimmelstiel-Wilson's nodular lesions with or without microaneurysm and mesangiolysis, eventually leading to glomerulosclerosis, in advanced stages.

Abnormal angiogenesis has long been implicated in the morphology and pathophysiology of diabetic nephropathy. Initially, new blood vessel formation in glomeruli representing aberrant angiogenesis was reported in patients with type 1 diabetes [22]. Other groups then reported similar findings in patients with type 2 diabetes [23, 24]. Such abnormal blood vessels were observed in the glomerular tuft area, Bowman's capsule, and the glomerular vascular pole [22, 25]. An interesting study using computer-aided reconstruction of three-dimensional images in patients with diabetic nephropathy demonstrated that these abnormal vessels are anastomosed to lobular structure of the intraglomerular capillary network and that the distal end of the vessels is anastomosed to the peritubular capillary [26]. In animal studies, type 1 and type 2 diabetic rodent models showed formation of new glomerular capillaries and the elongation of preexisting capillaries [27, 28], which are similar findings to those observed in human diabetic nephropathy. These abnormal new vessels have been considered to be associated with the increased glomerular filtration surface, leading to glomerular hypertrophy and hyperfiltration in the early stages of diabetic nephropathy.

VEGF-A expression is likely to be associated with the formation of abnormal new vessels. In an experimental diabetic model, renal protein and mRNA levels of VEGF-A and VEGFR-2 were upregulated in the early stage, and these increased levels persisted in the late stage [29]. Similarly, plasma and urinary levels of VEGF-A were elevated in patients with diabetic nephropathy [30, 31]. Recent clinical study revealed that increased circulating VEGF-A in type 2 diabetic patients was correlated with glycemic control, high-sensitive C-reactive protein, and albuminuria, suggesting the role of VEGF-A as a biomarker of inflammation and nephropathy in diabetes [32]. Another study involving type 2 diabetic patients showed a significant correlation between circulating VEGF-A and serum levels of hypoxia-inducible factor-1*α* (HIF-1*α*) and insulin-like growth factor-1 (IGF-1), which are considered to be involved in the pathogenesis of diabetic nephropathy [33]. However, some results of glomerular VEGF-A expression in human diabetic nephropathy have been controversial. Immunohistochemical analysis on renal biopsies revealed that glomerular VEGF-A was increased in the early stage of diabetic nephropathy [34], whereas oligonucleotide microarray analysis on human kidneys demonstrated that glomerular VEGF-A mRNA levels were decreased in patients with diabetic nephropathy [35]. Considering the fact that the decreased VEGF-A was observed in severely injured glomeruli with reduced podocyte markers in the latter study, glomerular VEGF-A levels might in fact decrease in the advanced stage of diabetic nephropathy. Indeed, glomerular expression of VEGF-A was shown to be decreased in sclerotic areas and nodular lesions of human diabetic nephropathy [36]. Therefore, increased glomerular VEGF-A in the early stage is probably involved in the characteristic alterations, including abnormal angiogenesis, while decreased glomerular VEGF-A in the later stage of the disease may promote glomerular scarring.

## 4. Biology of Glomerular VEGF

VEGF-A plays important role not only in maintaining glomerular capillary structure but also in repairing it following glomerular endothelial injuries [37, 38]. VEGF-A is constitutively expressed in podocytes and, to lesser extent, in tubular epithelial cells [39]. Since the expression of VEGFR-2 is localized to endothelial cells in glomeruli [40], there are important interactions that occur between podocytes and glomerular endothelial cells via the VEGF-A-VEGFR-2 axis. Indeed, VEGF-A is considered to be transported via diffusion across the glomerular basement membrane from podocytes to endothelial cells, against the flow of glomerular filtration [41].

**Figure 1:**
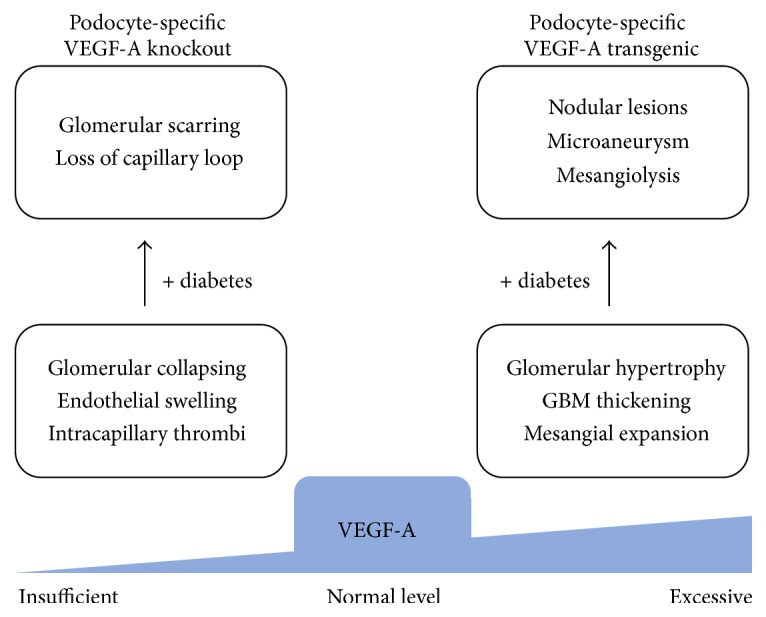
Histological alterations of glomeruli associated with excessive (“too much”) or insufficient (“too little”) glomerular vascular endothelial growth factor (VEGF)-A. In conditionally podocyte-specific VEGF-A transgenic mice, VEGF-A-overexpressing glomeruli become hypertrophic with glomerular basement membrane (GBM) thickening and mesangial expansion, similar to early stages of diabetic glomerulopathy [45]. Induction of diabetes in these transgenic mice results in Kimmelstiel-Wilson-like nodular lesions, microaneurysms, and mesangiolysis in the glomeruli [46], which are observed in advanced diabetic nephropathy. In contrast, conditionally podocyte-specific VEGF-A deficient mice show glomerular capillary thrombi and obliterated capillary loops with swollen endothelial cells, consistent with the findings of renal thrombotic microangiopathy [44]. Diabetes accelerates the dropout of glomerular capillaries in this conditional knockout mice, leading to glomerulosclerosis [47].

Pivotal roles of VEGF-A expression in podocytes were demonstrated via a series of elegant experiments using genetically modified mice. Podocyte-specific heterozygous VEGF-A deficient mice showed proteinuria and glomerular endothelial injury similar to preeclampsia, and podocyte-specific VEGF-A_164_-overexpressing mice showed marked collapsing glomerulopathy [42]. In another report, transgenic rabbits that express human VEGF-A_165_ in both the kidneys and liver under the control of a *α*1-antitrypsin promoter also exhibited progressive proteinuria and renal dysfunction with prominent glomerular capillary proliferation and podocyte hypertrophy at the early stage and then glomerulosclerosis and tuft collapse at the later stage [43]. More recently, the importance of glomerular VEGF-A expression in an adult kidney was examined using conditional gene expression, or the deletion technique. Eremina et al. conditionally deleted VEGF-A gene from podocytes in adult mice and observed increased proteinuria as well as intracapillary thrombi and obliterated capillary loops with swollen endothelial cells, resembling renal thrombotic microangiopathy [44]. On the other hand, Veron et al. induced podocyte-specific overexpression of VEGF-A in adult transgenic mice and observed proteinuria, glomerulomegaly, glomerular basement membrane thickening, mesangial expansion, and podocyte effacement [45]. Taken together, these results suggested that a “normal” level of VEGF-A is essential for maintaining the glomerular capillary structure, including the glomerular filtration barrier in the adult kidneys, and both too much and too little VEGF-A in glomeruli can lead to significant renal pathology (Figure 1).

In diabetic nephropathy, VEGF-A is likely a crucial mediator, according to a number of publications. As described above, increased glomerular expression of VEGF-A is seen in patients with diabetic nephropathy as well as in its animal models. In addition, human VEGF-A_165_ transgenic rabbits developed microaneurysms [43], while podocyte-specific VEGF-A-overexpressing mice showed glomerular basement membrane thickening and mesangial expansion [45], both of which are similar to the histology of diabetic nephropathy. Furthermore, selective VEGFR-2 stimulation by overexpression of the mutant form of VEGF-A binding only to VEGFR-2 in mice resulted in mesangial matrix expansion with endothelial cell proliferation [48]. These findings suggest that increased glomerular expression of VEGF-A is sufficient to cause early glomerular alterations in diabetic nephropathy. However, hyperglycemia is necessary to develop advanced lesions. Veron et al. induced diabetes in podocyte-specific conditional VEGF-A_164_ transgenic mice and observed massive proteinuria as well as Kimmelstiel-Wilson-like nodular glomerulosclerosis, microaneurysms, and mesangiolysis in the glomeruli of the mice [46], which was consistent with advanced diabetic glomerulopathy (Figure 1).

Such synergistic effects of hyperglycemia and increased VEGF-A in diabetic glomerulopathy may be explained by the unique hypothesis of “uncoupling of VEGF-A with nitric oxide (NO)” [49, 50]. Normally, VEGF-A stimulates endothelial NO release, and NO is required for the actions of VEGF-A on endothelial cells. When hyperglycemia impairs normal endothelial function and reduces NO production, elevated levels of glomerular VEGF-A noted in diabetes could exert deleterious effects on endothelial cells, leading to diabetic glomerulopathy. Indeed, endothelial NO synthase- (eNOS-) deficient mice with streptozotocin-induced hyperglycemia (type 1 diabetic model) or those crossbred with obese db/db mice (type 2 diabetic model) both exhibited massive proteinuria and glomerular alterations identical to human advanced diabetic nephropathy [51, 52]. Diabetic eNOS deficient mice also developed profound podocyte injuries, possibly due to the impairment of crosstalk between glomerular endothelial cells and podocytes [53]. Similarly, db/db mice with VEGFR-1 inhibition, which enhanced VEGFR-2 activity, showed prominent albuminuria and mesangial expansion together with a loss of podocytes and endothelial cells [54]. Furthermore, even without hyperglycemia, podocyte-specific VEGF-A overexpression in eNOS-null mice resulted in nodular glomerulosclerosis, mesangiolysis, and microaneurysms that were associated with massive proteinuria [55]. Conversely, NO donor nicorandil ameliorated proteinuria and glomerular pathology, including podocyte injury, in diabetic eNOS knockout mice [56]. These findings emphasize the advantage of specific therapies targeting uncoupling of VEGF-A with NO in diabetic nephropathy by suppressing increased glomerular VEGF-A or supplementing endothelial NO.

VEGF-A_165_b is also expressed in immature podocytes, but the expression is lower in matured glomeruli [57]. Recently, VEGF-A_165_b was shown to be upregulated in renal cortical tissues taken from patients with early diabetic nephropathy [58]. Podocyte-specific overexpression of VEGF-A_165_b in diabetic mice resulted in the amelioration of diabetic glomerulopathy [58], suggesting the protective role of increased VEGF-A_165_b in diabetic nephropathy.

Unlike VEGF-A, involvement of the other VEGF family members in diabetic glomerulopathy has not been well-established. It has been shown that VEGF-B is mainly expressed in renal medullary tubular cells, but not in glomeruli, whereas expression of its receptor VEGFR-1 was found in endothelial cells [59]. However, a recent report did demonstrate that VEGF-B inhibition suppressed histological alterations and renal dysfunction in diabetic mice [60]. Although VEGFR-1 inhibition results in the exacerbation of diabetic glomerulopathy as above [54], VEGF-B inhibition was found to reduce lipotoxicity and improve insulin-resistance in podocytes in this study [60], suggesting that VEGF-B is probably involved in diabetic glomerulopathy through nonangiogenic mechanisms.

## 5. Anti-VEGF Therapies for Diabetic Nephropathy

As described above, the fact that the increased expression of VEGF-A in podocytes associated with hyperglycemia leads to characteristic glomerular alterations provides the rationale for anti-VEGF therapy against diabetic nephropathy. The landmark experiments revealed that the administration of neutralizing monoclonal anti-VEGF antibodies to type 1 and type 2 diabetic animals decreased albuminuria and glomerular hypertrophy [61, 62], indicating the efficacy of anti-VEGF therapy against diabetic nephropathy. Then, SU5416, a pan-VEGF receptor tyrosine kinase inhibitor, was also reported to reduce albuminuria in type 2 diabetic mice [63].

However, there have been emerging concerns about anti-VEGF therapy in humans. Soon after introduction of bevacizumab, a humanized monoclonal anti-VEGF antibody, in clinical practice to prevent cancer growth and metastasis, proteinuria and hypertension were reported to occur as common complications [64]. These clinical findings were subsequently reported in patients treated with multitargeted tyrosine kinase inhibitors (TKIs), small molecules that inhibit VEGFR intracellular intrinsic kinases, such as sunitinib and sorafenib [65]. Based on the observation of decreased urinary nitrite/nitrate excretion and serum levels of NO metabolites in patients treated with VEGF inhibitors [66], hypertension induced by anti-VEGF antibody may be involved in the disruption of the VEGF-A-endothelial NO axis as noted above. Proteinuria is probably caused as a result of impaired interaction between podocytes and glomerular endothelial cells. Eremina et al. first reported that bevacizumab treatment in some cancer patients led to a renal pathology of glomerular endothelial swelling, red blood cell fragmentation, and intracapillary thrombi, which were characteristics of thrombotic microangiopathy, and subsequently reproduced these findings in podocyte-specific VEGF-A-deficient mice as above [44]. Recently, the novel role of VEGF-A in the kidneys was revealed as a potential mechanism underlying bevacizumab-related renal thrombotic microangiopathy. VEGF-A inhibition decreased the renal level of inhibitory complement factor H (CFH), in which genetic variants were known to be features of hereditary thrombotic microangiopathy, suggesting that VEGF-A is involved in local regulation of the complement system [67]. Therefore, anti-VEGF antibody therapy for diabetic nephropathy needs to eliminate only the “excess” glomerular VEGF-A but must not lower it to a subnormal level. However, considering that diabetes induces endothelial dysfunction and reduces NO bioavailability, administration of anti-VEGF antibodies in the diabetic condition is likely to result in proteinuria and renal dysfunction. Indeed, diabetes was a major risk factor for proteinuria in bevacizumab-treated patients [68, 69]. Furthermore, in conditionally podocyte-specific VEGF-A-deficient mice, diabetes accelerated proteinuria and apoptosis of glomerular endothelial cells, leading to profound glomerular scarring [47] (Figure 1). At present, anti-VEGF antibody or TKIs therapy for diabetic nephropathy is not warranted.

There are several endogenous antiangiogenic systems to prevent excessive angiogenesis in the body (Table 1). Important information regarding such systems has been derived from understanding the pathogenesis of preeclampsia. One of the endogenous antiangiogenic factors involved in preeclampsia is soluble fms-like tyrosine kinase (sFlt-1). sFlt-1 is a soluble form of VEGFR-1 capable of binding to VEGF-A, VEGF-B, and PlGF and acts as a potent VEGF antagonist. Conditionally podocyte-specific overexpression of sFlt-1 in mice ameliorated diabetic glomerulopathy as well as albuminuria [70], and adeno-associated virus transferred sFlt-1 overexpression in db/db mice resulted in reduced albuminuria and improved podocyte injury [71]. Furthermore, podocyte-specific overexpression of angiopoietin-1, which was a regulator for vascular stabilization, was recently shown to prevent albuminuria as well as glomerular endothelial proliferation with elevated levels of sFlt-1 [72]. However, in an earlier report, the intravenous injection of sFlt-1 in mice induced proteinuria [73]. In addition, adenoviral transfer of sFlt-1 in mice induced proteinuria and caused glomerular endotheliosis similar to VEGF-A-deficient glomeruli [74]. Thus, sFlt-1 therapy for diabetic nephropathy potentially has the same concerns as the use of anti-VEGF antibodies.

VEGF-A_165_b acts as a weaker VEGFR-2 agonist (Table 1). Recently, therapeutic effects of recombinant human VEGF-A_165_b in type 1 and type 2 diabetic mice were reported. In this study, VEGF-A_165_b ameliorated albuminuria and glomerular basement membrane thickening and normalized VEGFR-2-mediated glomerular permeability [58]. In addition, VEGF-A_165_b restored glomerular endothelial glycocalyx, which constitutes a barrier to glomerular permeability and is injured by diabetes [58], suggesting that VEGF-A_165_b possibly provides endothelial protection in diabetic nephropathy. Interestingly, podocyte-specific overexpression of VEGF-A_165_b in mice reduced endothelial fenestrations [75] but did not result in increased urinary albumin excretion and renal thrombotic microangiopathy [58]. Moreover, VEGF-A_165_b was reported to protect endothelial cells from cytotoxicity induced by serum starvation in vitro [76]. As such, VEGF-A_165_b may be a promising therapy for those diagnosed as having diabetic nephropathy. However, given the weak agonist activity of VEGF-A_165_b for VEGFR-2, an appropriate dosage regimen would need to be addressed in the future studies.

## 6. Alternative Antiangiogenic Therapies

Although anti-VEGF therapy could lead to the potential renal adverse effects in cancer treatment, a much lower dose regimen may be a possible option for use in those with diabetic nephropathy. However, the therapeutic efficacy of such low-dose regimen has not yet been investigated in diabetic animal experiments. Novel drugs targeting intracellular downstream signaling pathways of VEGFR-2 could be effective and safe therapeutic options in the future. Considering the limitations of current anti-VEGF therapies, alternative antiangiogenic therapies that do not serve as direct inhibitors of VEGF-A may have potential benefits for the treatment of diabetic nephropathy. Some extracellular matrix protein fragments are known to act as circulating endogenous antiangiogenic factors and have antitumor efficacies (Table 1). These antiangiogenic factors interfere with VEGF-A signaling processes but do not directly antagonize VEGF-A.

Tumstatin is derived from a type IV collagen *α*3 chain and inhibits pathological angiogenesis via suppression of endothelial cell proliferation [77]. It binds to the *α*v*β*3 integrin of endothelial cells [78]. The antiangiogenic activity of tumstatin is considered to be based on the inhibition of focal adhesion kinase (FAK), PI3 kinase, protein kinase B (PKB/Akt), and the mammalian target of rapamycin (mTOR) in endothelial cells [79]. The therapeutic effects of tumstatin-derived peptide on diabetic nephropathy in a type 1 diabetic mouse model were examined [80]. In the study, tumstatin peptide significantly suppressed albuminuria and glomerular histological alterations as well as increased the number of glomerular capillaries in diabetic mice. Tumstatin peptide also significantly suppressed the increase in renal VEGF-A and VEGFR-2 induced by diabetes.

Endostatin is derived from type XVIII collagen and possesses potent inhibitory effects on tumor growth [81]. It also inhibits VEGF-induced endothelial cell proliferation, migration, and tube formation in vitro [82]. Endostatin interacts with *α*5*β*1 integrin, leading to the inhibition of FAK and subsequent inhibition of mitogen-activated protein kinases (MAPKs) [83]. The therapeutic potential of endostatin in nonneoplastic disorders with angiogenic processes has been reported [84–86]. In type 1 diabetic mice, endostatin peptides significantly suppressed albuminuria and histological alterations [87]. They also significantly suppressed the expansion of glomerular capillary area and increased VEGF-A and VEGFR-2 in diabetic mice. Because *α*5*β*1 integrin is localized to endothelial cells in glomeruli and upregulated in diabetes [87], endostatin primarily acted on the glomerular endothelial cells. On the other hand, recent clinical study revealed that type 2 diabetic patients with nephropathy had a higher circulating level of endostatin, indicating the clinical usefulness of endostatin as a risk marker of diabetic nephropathy [88].

Unlike tumstatin and endostatin, angiostatin is a proteolytic fragment of plasminogen and inhibits tumor neovascularization [89]. Adenoviral delivery of angiostatin significantly ameliorated albuminuria and glomerular hypertrophy in a type 1 diabetic rat model [90]. It also suppressed the increased expression of VEGF-A in diabetic kidneys. In another report, however, adenoviral overexpression of angiostatin in a remnant kidney model resulted in a reduction in peritubular capillary density [91].

Taken together, these results suggest the therapeutic potential of tumstatin, endostatin, and angiostatin in diabetic nephropathy. However, their therapeutic effects may not necessarily be associated with antiangiogenic properties. For example, as *α*v*β*3 integrin, the receptor for tumstatin, is heavily expressed in podocytes [92], the primary target for tumstatin may not be endothelial cells but podocytes. Endostatin suppressed glomerular VEGF-A mainly produced by podocytes in diabetic mice [87], and thus it might act on podocytes rather than endothelial cells. Therefore, the mechanisms of their therapeutic efficacy in diabetic nephropathy, as well as antiangiogenic effects on endothelial cells, have not yet been convincingly and fully elucidated. This fact limits the clinical use of these fragments as antiangiogenic drugs for diabetic nephropathy. Among these fragments, endostatin has been already introduced in clinical practice as an anticancer drug to be combined with definitive chemotherapy. Recombinant human endostatin (Endostar®, Nanjing NingQi Medicine Science and Technology Co., Ltd., Nanjing, China) was developed and approved for lung cancer in China. Notably, recombinant human endostatin did not result in hypertension and proteinuria [93, 94] or did not significantly elevate the incidence of proteinuria [95] in clinical trials. Recombinant human angiostatin is undergoing clinical trial for anticancer efficacy in patients with lung cancer, but this trial has not been completed. Tumstatin has not been considered in clinical trials yet.

## 7. Vasohibin-1 as a Novel Therapeutic Agent

A novel endogenous angiogenesis inhibitor, Vasohibin-1 (VASH1), was identified in a microarray analysis performed to explore genes upregulated by VEGF-A in endothelial cells [96]. The gene for human VASH1 is located on chromosome 14q24.3 and consists of seven exons, which is highly conserved in vertebrates. Human VASH1 protein is composed of 365 amino acids and serves as an endothelial cell-derived negative feedback regulator of angiogenesis; that is, it is upregulated in endothelial cells in response to proangiogenic stimuli and acts on endothelial cells to inhibit its activation (Table 1). Functional analysis revealed that some basic amino acid residues at the C-terminus of VASH1 were important for heparin binding and antiangiogenic activity [97]. VASH1 is known to be a secretory protein, and coexpression of small vasohibin-binding protein (SVBP) is required for the secretion and antiangiogenic activity of VASH1 [98]. An anticancer effect of VASH1 through its inhibition of tumor angiogenesis has been confirmed in several reports [96, 99, 100]. Although this protein was shown to induce prolyl hydroxylase-mediated degradation of hypoxia-inducible factor-1*α* [101], the precise mechanism for its antiangiogenic activity remains to be elucidated. The receptor(s) for VASH1 on endothelial cells and its intracellular signaling pathway have not yet been detected. Notably, VASH1 does not induce apoptosis but rather promotes survival in endothelial cells, unlike other antiangiogenic factors (Table 1). In vitro analyses, knockdown of VASH1 induced premature senescence of endothelial cells and those cells became highly vulnerable to death caused by cellular stress [102]. In contrast, the overexpression of VASH1 made endothelial cells resistant to premature senescence and stress-induced cell death with augmented expression of superoxide dismutase 2 (SOD2) and sirtuin 1 (Sirt1) [102], suggesting that VASH1 improved the stress tolerance of endothelial cells. In addition, adenoviral transfer of human VASH1 gene to mice inoculated with Lewis lung carcinoma cells not only inhibited tumor angiogenesis but also matured remaining tumor vessels [99]. Such VASH1-induced vessel maturation led to enhanced anticancer effect of cisplatin, probably due to the improved delivery of the agent to cancer cells. Therefore, VASH1 inhibits VEGF-A-induced “excessive” angiogenic response in endothelial cells, along with enhancing the tolerance to cellular stresses and prolonging its survival, leading to the protection and stabilization of vessels.

Increased expression of VASH1 has been observed in various human malignancies and correlated with poor prognosis [103–105]. Such malignancies possibly upregulate VASH1 in order to suppress the growth and metastasis. We recently reported the clinical significance of VASH1 in patients with kidney diseases. First, the correlation between plasma and urinary VASH1 and clinical parameters was evaluated [106]. Plasma levels of VASH1 were inversely correlated with age and blood pressure. Moreover, it was found that elevated plasma and urinary levels of VASH1 predicted worse renal prognosis in patients with kidney diseases. Second, the renal distribution of VASH1 in renal biopsy specimens taken from patients with kidney diseases was evaluated [107]. VASH1 was observed in endothelial cells and in glomerular crescentic lesions and interstitial inflammatory cells. The number of VASH1-positive cells in the glomeruli was correlated with glomerular VEGFR-2-positive area and crescent formation. These results suggest that increased systemic and renal expression of VASH1 is associated with the progression of kidney diseases. Given the same tendency that VASH1 expression is associated with poor prognosis of cancer [103–105], VASH1 may be upregulated in kidney diseases in order to counter cellular stress such as local inflammation.

Thus far, the therapeutic efficacies of VASH1 on several nonneoplastic disorders associated with angiogenesis, such as atherosclerosis, macular degeneration, and bronchiolitis obliterans, have been reported [108–110]. The potential role of VASH1 as a biomarker for rheumatoid arthritis was also demonstrated [111]. Based on both the antiangiogenic and the endothelial protective effects of VASH1, we evaluated the therapeutic effects of VASH1 in type 1 and type 2 diabetic nephropathy mouse models [112, 113]. These mice were given intravenous injections of adenoviral vectors encoding human VASH1 (Ad-VASH1) every two weeks. In both mouse models, VASH1 overexpression significantly ameliorated glomerular hypertrophy, glomerular hyperfiltration, and albuminuria and also expanded glomerular endothelial area in the diabetic mice. Diabetes-induced mesangial type IV collagen accumulation and glomerular monocytes infiltration were also suppressed by treatment with Ad-VASH1. In type 1 (streptozotocin-induced) diabetic mice, enhanced phosphorylation of VEGFR-2 was prevented in kidneys treated with Ad-VASH1. Recombinant human VASH1 (rhVASH1) also prevented the phosphorylation of VEGFR-2 induced by high glucose levels in cultured glomerular endothelial cells in a dose-dependent manner [112]. Thus, VASH1 inhibits the excess angiogenic response in diabetic glomeruli by preventing activation of VEGFR-2. In addition, rhVASH1 suppressed the increased transforming growth factor-*β* (TGF-*β*) and monocyte chemotactic protein-1 (MCP-1) induced by high glucose level in cultured mesangial cells. In type 2 diabetic (db/db) mice, adenoviral overexpression of VASH1 relieved a diabetes-induced podocyte injury. Treatment with rhVASH1 also restored the expression of epithelial markers and prevented the expression of mesenchymal markers in cultured podocytes. Therefore, VASH1 was likely to directly act on mesangial cells and podocytes in diabetic glomeruli.

Furthermore, the roles of endogenous VASH1 in diabetic nephropathy was underscored by our recent report using VASH1 heterozygous knockout (VASH1^+/−^) mice [114]. In the recent study, streptozotocin-induced type 1 diabetic VASH1^+/−^ mice exhibited increased albuminuria, glomerular hypertrophy, and mesangial matrix accumulation and decreased slit diaphragm density. Glomerular CD31-positive area and renal VEGF-A expression were enhanced in the diabetic VASH1^+/−^ mice as compared with diabetic wild type mice. Glomerular monocytes infiltration and nuclear translocation of activated NF-*κ*B were also exacerbated in the diabetic VASH1^+/−^ mice [114]. Thus, endogenous VASH1 probably prevents both angiogenic and inflammatory responses in diabetic glomeruli, as similar anti-inflammatory effect of endogenous VASH1 was also confirmed in a unilateral ureteral obstruction model [115].

Taken together, these results suggest the therapeutic potential of VASH1 for early diabetic nephropathy through suppressing excessive angiogenic response in endothelial cells and protecting mesangial cells and podocytes from diabetic insult. Since VASH1 increases the stress tolerance of endothelial cells and promotes their survival [116], VASH1 therapy for diabetic nephropathy should not only eliminate the risk of endothelial injury as shown by anti-VEGF antibodies but also add the benefit of protecting endothelial cells from hyperglycemia.

## 8. Conclusion

Abnormal angiogenesis is involved in the pathogenesis of diabetic nephropathy, and VEGF-A is considered to be the most important mediator. Although anti-VEGF therapy is a rational approach, recent evidence suggests that the suppression of VEGF-A to subnormal levels results in renal thrombotic microangiopathy, with leading to glomerular scarring especially in diabetic conditions with endothelial dysfunction. VEGF-A_165_b has the potential to be a therapeutic agent. Matrix-derived antiangiogenic factors such as endostatin are likely to suppress increased angiogenic response in diabetic glomeruli without excessive VEGF-A inhibition. VASH1 possesses both antiangiogenic and endothelial protective efficacies, and, thus, it will likely become a favorable candidate for antiangiogenic therapy. Based on the experimental evidence, a novel antiangiogenic therapy that prevents abnormal angiogenic response in glomeruli but does not induces endothelial injury should be a promising approach for the treatment of diabetic nephropathy.

## Figures and Tables

**Table 1:** Candidates of endogenous antiangiogenic factors as therapeutic agents for diabetic nephropathy.

	Description	Target molecule	Effect on ECs	Clinical use
sFlt-1	Soluble form of VEGFR-1 that binds to circulating VEGF and prevents it from binding to VEGFR-2.	VEGF	Apoptosis	None
VEGF-A_165_b	Inhibitory VEGF-A splice variant which induces insufficient phosphorylation of VEGFR-2.	VEGFR-2	Survival?^*∗*^	None
Tumstatin	Protein fragment cleaved from type IV collagen that binds to endothelium via integrin and inhibits protein synthesis.	*α*v*β*3-integrin	Apoptosis	None
Endostatin	Protein fragment cleaved from type XVIII collagen which acts on endothelium to suppress cell cycle genes and antiapoptotic genes.	*α*5*β*1-integrin (glypicans, VEGFR-2)	Apoptosis	Available in China
Angiostatin	Protein fragment cleaved from plasminogen which binds to potentially many proteins to induce its apoptosis.	Angiomotin and others^*∗∗*^	Apoptosis	Under trial
Vasohibin-1	Endothelium-derived protein that causes negative feedback response in endothelial cells stimulated by VEGF-A and promotes its survival by inducing SOD2 and Sirt1.	Unknown	Survival	None

ECs, endothelial cells; sFlt-1, soluble fms-like tyrosine kinase-1; VEGF, vascular endothelial growth factor; VEGFR-2, VEGF receptor-2; SOD2, superoxide dismutase-2; Sirt1, sirtuin-1. ^*∗*^VEGF -A_165_b may attenuate endothelial survival effect of VEGF-A_165_, but endothelial protective effect was also reported (see text). ^*∗∗*^Other representative molecules include surface ATP synthase, NG2 proteoglycan, c-Met, and annexin II.
